# Momentum-Space Decoherence of Distinguishable and Identical Particles in the Caldeira–Leggett Formalism

**DOI:** 10.3390/e23111469

**Published:** 2021-11-07

**Authors:** Z. Khani, S. V. Mousavi, S. Miret-Artés

**Affiliations:** 1 Department of Physics, University of Qom, Ghadir Blvd., Qom 371614-6611, Iran; z.khani@stu.qom.ac.ir (Z.K.); vmousavi@qom.ac.ir (S.V.M.); 2 Instituto de Física Fundamental, Consejo Superior de Investigaciones Científicas, Serrano 123, 28006 Madrid, Spain

**Keywords:** decoherence, Caldeira–Leggett formalism, momentum space, stretched Gaussian wave packet, cat state, bosons, fermions

## Abstract

In this work, momentum-space decoherence using minimum and nonminimum-uncertainty-product (stretched) Gaussian wave packets in the framework of Caldeira–Leggett formalism and under the presence of a linear potential is studied. As a dimensionless measure of decoherence, purity, a quantity appearing in the definition of the *linear entropy*, is studied taking into account the role of the stretching parameter. Special emphasis is on the open dynamics of the well-known cat states and bosons and fermions compared to distinguishable particles. For the cat state, while the stretching parameter speeds up the decoherence, the external linear potential strength does not affect the decoherence time; only the interference pattern is shifted. Furthermore, the interference pattern is not observed for minimum-uncertainty-product-Gaussian wave packets in the momentum space. Concerning bosons and fermions, the question we have addressed is how the symmetry of the wave functions of indistinguishable particles is manifested in the decoherence process, which is understood here as the loss of being indistinguishable due to the gradual emergence of classical statistics with time. We have observed that the initial bunching and anti-bunching character of bosons and fermions, respectively, in the momentum space are not preserved as a function of the environmental parameters, temperature, and damping constant. However, fermionic distributions are slightly broader than the distinguishable ones and these similar to the bosonic distributions. This general behavior could be interpreted as a residual reminder of the symmetry of the wave functions in the momentum space for this open dynamics.

## 1. Introduction

Decoherence is a crucial process in order to better understand the emergence of classical behavior in the quantum dynamics of physical systems [1,2,3,4]. This process arises when the physical system of interest interacts with an apparatus to carry out a measurement or when it is immersed in a given environment. The theory of open quantum systems is the natural framework to carry out these kinds of studies and has been widely developed from quite different approaches and published in several books [5,6,7,8,9,10,11,12]. Within the theoretical methods working with wave functions instead of reduced density matrix, one can find some approaches within the so-called Caldirola–Kanai and Scrödinger–Langevin frameworks [13,14,15,16]. Both approaches are not following the system-plus-environment model but effective time dependent Hamiltonians and nonlinear Schrödinger equations, respectively. Recently, interference and diffraction of identical spinless particles in one slit problems [17] have been analyzed.

In this work, we are going to focus on the so-called Caldeira–Leggett (CL) formalism [11,18]. This formalism is based on the reduced density matrix once one carries out the integration over the environmental degrees of freedom. As is well-known, the diagonal matrix elements give probabilities and off-diagonal matrix elements are called coherences. In the decoherence process, these off-diagonal elements go to zero more or less rapidly depending on the parameters characterizing the environment—usually, damping constant and temperature. Most of the studies involving quantum decoherence are being carried out in the configuration space and very few in the momentum space. Venugopalan [19] studied the decoherence of a *free single* minimum-uncertainty-product Gaussian wavepacket in the CL formalism within the context of measurement processes both in position and momentum spaces. This study revealed that the emergent *preferred basis* selected by the environment is the momentum basis. By considering a cat state, decoherence *without dissipation* has been studied in phase space [20]. To this end, these authors considered the quantum system in thermal equilibrium and assumed a weak interaction with the environment in a way that dissipation could be neglected. Then, from principles of statistical mechanics, the corresponding probability distribution were obtained by averaging over a thermal distribution of velocities. Furthermore, the Wigner phase space distribution function was also obtained and the destruction of the interference term was studied as a function of time. Decoherence was claimed not to occur in momentum and phase space. More recently, decoherence in momentum space has been studied in the context of suppression of quantum-mechanical reflection [21] using a master equation resembling the CL equation [11,18] in the negligible dissipation limit; and for a non-relativistic charged particle described by a wave packet under the presence of linear interaction with the electromagnetic field in equilibrium at a certain temperature [22]. Recently, in the chemical physics community, studies about purity are also found questioning this quantity as a measure of decoherence in the dynamics of quantum dissipative systems [23,24,25].

The central goal of this work is to show how decoherence affects the open dynamics of cat states and identical spinless (bosons and fermions) particles within the momentum representation, far less investigated than in the configuration space [26]. For cat states, while the stretching parameter speeds up the decoherence, the external linear potential strength does not affect the decoherence time; only the interference pattern is shifted. Furthermore, the interference pattern is not observed for minimum-uncertainty-product-Gaussian wave packets in the momentum space. Purity, a quantity appeared in the definition of the *linear entropy*, and its relation to coherence length is studied taking into account the role of the stretching parameter. The next question is how the symmetry of the corresponding wave functions is manifested in the decoherence process. This process is understood here as the loss of the indistinguishable character of those particles due to the gradual emergence of classical statistics with time. In particular, the well-known bunching and anti-bunching properties of bosons and fermions, respectively, when minimum and non-minimum-uncertainty-product Gaussian wavepackets are used is considered as a function of the environmental parameters, temperature, and damping constant. We have observed that the symmetry of the initial distribution is not preserved in the time evolution of the corresponding wave functions. However, fermionic distributions are slightly broader than the distinguishable ones and these are similar to the corresponding bosonic distributions. This could be interpreted as a residual reminder of the bunching and anti-bunching character of the initial distributions in the momentum space but washing them out when increasing the damping constant and temperature. This general behavior has also been confirmed by carrying out a different theoretical analysis from the single-particle probability. Finally, an indirect manifestation of these properties for bosons and fermions have also been observed when considering the so-called simultaneous detection probability.

This paper is organized as follows: in Section 2, the CL master equation in the momentum representation is briefly introduced. In Section 3, open dynamics and decoherence of minimum and non-minimum-uncertainty-product Gaussian wavepackets are analyzed for cat states and under the presence of a linear potential. Then, open dynamics of two identical spinless particles (bosons and fermions) are analyzed in Section 4. In Section 5, results, discussion, and some concluding remarks are presented.

## 2. The Caldeira–Leggett Master Equation in the Momentum Representation

In the context of open quantum systems and considering the reservoir as a set of non-interacting oscillators, Caldeira and Leggett obtained the well-known master equation [11,18]

(1) $$\begin{array}{ccc} \frac{\partial \hat{\rho }}{\partial t} & = & \frac{1}{i\hbar }\left[{\hat{H}}_{0},\hat{\rho }\right]+\frac{\gamma }{i\hbar }\left[\hat{x},\left\{\hat{p},\hat{\rho }\right\}\right]-\frac{D}{\hbar^{2}}\left[\hat{x},\left[\hat{x},\hat{\rho }\right]\right] \end{array}$$

for the reduced density matrix of the system where γ is the damping constant or dissipation rate, and $D=2m\gamma k_{B}T$ plays the role of the diffusion coefficient with *m* the mass of particles; $k_{B}$ and *T* being Boltzmann’s constant and the environment temperature, respectively. The Hamiltonian ${\hat{H}}_{0}$ is given by

(2) $$\begin{array}{ccc} {\hat{H}}_{0} & = & \frac{{\hat{p}}^{2}}{2m}+\hat{V}. \end{array}$$

Equation (1) in the momentum representation for an external potential $\hat{V}=V\left(\hat{x},\hat{p}\right)$ reads as

(3) $$\begin{array}{ccc} \frac{\partial }{\partial t}\rho \left(p,p^{\prime },t\right) & = & [-\frac{i}{2m\hbar }\left(p^{2}-p^{\prime 2}\right)+\frac{V\left(i\hbar \frac{\partial }{\partial p},p\right)-V\left(-i\hbar \frac{\partial }{\partial p^{\prime }},p^{\prime }\right)}{i\hbar }\\ & & +\gamma \left(\frac{\partial }{\partial p}+\frac{\partial }{\partial p^{\prime }}\right)\left(p+p^{\prime }\right)+D{\left(\frac{\partial }{\partial p}+\frac{\partial }{\partial p^{\prime }}\right)}^{2}]\rho \left(p,p^{\prime },t\right) \end{array}$$

where the off-diagonal matrix elements are $\rho \left(p,p^{\prime },t\right)=\langle p|\hat{\rho }|p^{\prime }\rangle$ and known as coherences. In the center of mass and relative coordinates,

{(4a)u=p+p′2(4b)v=p−p′

Equation (3) for the external linear potential $\hat{V}=mg\hat{x}$ can be expressed as

(5) $$\begin{array}{ccc} \frac{\partial }{\partial t}\rho \left(u,v,t\right)+\frac{\partial }{\partial u}j\left(u,v,t\right)+\frac{i}{m\hbar }uv\rho \left(u,v,t\right) & = & 0. \end{array}$$

the current density matrix being

(6) $$\begin{array}{ccc} j(u,v,t) & = & -\left(mg+2\gamma u+D\frac{\partial }{\partial u}\right)\rho \left(u,v,t\right). \end{array}$$

As is known, when v=0, the diagonal elements of the density matrix give the probability density and the continuity equation is written as

(7) $$\begin{array}{ccc} \frac{\partial P(p,t)}{\partial t}+\frac{\partial J(p,t)}{\partial p} & = & 0, \end{array}$$

where P(p,t) and J(p,t) are the diagonal elements of ρ(u,v,t) and j(u,v,t), respectively.

## 3. Open Dynamics and Decoherence of Gaussian Wave Packets: The Cat State

Let us consider a linear potential given by $\hat{V}=mg\hat{x}$ for nonminimum-uncertainty-product or *stretched* Gaussian wave packets in the CL framework for two cases: the open dynamics of a single wave packet and afterwards the corresponding dynamics for a pure initial state consisting of superposition of two well separated wavepackets, a cat state.

### 3.1. A Single Gaussian Wave Packet in a Linear Potential

For a single Gaussian wave packet, the solution of Equation (5) can be easily found by assuming the Gaussian ansatz,

(8) $$\begin{array}{ccc} \rho (u,v,t) & = & \frac{1}{\sqrt{2\pi d_{2}\left(t\right)}}\exp\left[d_{0}\left(v,t\right)-\frac{{\left(u-d_{1}\left(v,t\right)\right)}^{2}}{4d_{2}\left(t\right)}\right] \end{array}$$

and, from Equation (6), one has that

(9) $$\begin{array}{ccc} j(u,v,t) & = & \left[-mg-2\gamma u+\frac{D}{2d_{2}\left(t\right)}\left(u-d_{1}\left(v,t\right)\right)\right]\rho \left(u,v,t\right). \end{array}$$

On the other hand, let us consider the initial state as the stretched Gaussian wave packet whose Fourier transform takes the form

(10) $$\begin{array}{ccc} \phi_{0}\left(p\right) & = & {\left(\frac{2}{\pi }\frac{\sigma_{0}^{2}}{\hbar^{2}}\right)}^{1/4}\exp\left[-\left(1+i\eta \right)\frac{{\left(p-p_{0}\right)}^{2}\sigma_{0}^{2}}{\hbar^{2}}-i\frac{\left(p-p_{0}\right)x_{0}}{\hbar }\right]. \end{array}$$

Here, $x_{0}$ and $p_{0}$ are the center and kick momentum, and η is the stretching parameter governing the position-space width, $\Delta x=\sigma_{0}\sqrt{1+\eta^{2}}$. Thus, the uncertainty product $\Delta x\Delta p=\frac{\hbar }{2}\sqrt{1+\eta^{2}}$ reaches the minimum value for η=0. With this in mind, the solution of Equation (8) reads

{(11a)d0(v,t)=−iℏxtv−(η2+1)σ022ℏ2+ητ(t)2mℏ+τ(t)28m2σ02−D3+e−4γt−4e−2γt−4γt16ℏ2m2γ3v2(11b)d1(v,t)=pt−iℏ4mσ02τ(t)+η2e−2γt+Dmℏτ(t)2v(11c)d2(t)=ℏ28σ02e−4γt+D1−e−4γt4γ

with

{(12a)xt=x0+p0mτ(t)+gτ(t)−t2γ(12b)pt=p0e−2γt−mgτ(t)(12c)τ(t)=1−e−2γt2γ.

Note that $x_{t}$ is the trajectory followed by a *classical* particle with mass *m* and initial velocity $p_{0}/m$ immersed in a viscid media with a damping constant γ and under the presence of a constant force field −mg; and $p_{t}=m{\dot{x}}_{t}$ [14]. By imposing the conditions v=0, the probability density (PD) and the probability current density (PCD) are expressed as

(13) $$\begin{array}{ccc} P(p,t) & = & \frac{1}{\sqrt{2\pi }w_{t}}\exp\left[-\frac{{\left(p-p_{t}\right)}^{2}}{2w_{t}^{2}}\right] \end{array}$$

(14) $$\begin{array}{ccc} J(p,t) & = & \left[-mg-2\gamma p+\frac{D}{w_{t}^{2}}\left(p-p_{t}\right)\right]P\left(p,t\right) \end{array}$$

with

(15) $$\begin{array}{ccc} w_{t} & = & \sqrt{2d_{2}\left(t\right)}=e^{-2\gamma t}\frac{\hbar }{2\sigma_{0}}\sqrt{1+D\frac{2\sigma_{0}^{2}}{\hbar^{2}\gamma }\left(e^{4\gamma t}-1\right)} \end{array}$$

being the width of the distribution function in momentum space. As Equations (13) and (14) clearly show, in the momentum representation, the stretching parameter η plays no role in the PD and PCD.

In the long time limit, γt≫1, only terms which are constant and/or depend linearly on time survive and one has that

{(16a)xt≈−g2γt,(16b)pt≈−mg2γ,(16c)d0(t)≈iℏg2γv−D4ℏ2m2γ2v2t,(16d)d1(t)≈−mg2γ−iD4mℏγ2v,(16e)d2(t)≈D4γ,

yielding

(17) $$\begin{array}{c} \rho \left(u,v,t\right)\approx \sqrt{\frac{\gamma }{\pi D}}\exp\left[-\frac{4\gamma^{2}u^{2}+4m\gamma gu+m^{2}g^{2}}{4\gamma D}+\left(\frac{i}{\hbar }\frac{g}{2\gamma }v-\frac{D}{4\hbar^{2}m^{2}\gamma^{2}}v^{2}\right)t\right] \end{array}$$

showing that the off-diagonal elements of the reduced density matrix, v≠0, decay exponentially with time. This allows us to define a time as

(18) $$\begin{array}{ccc} t_{d} & = & \frac{4\hbar^{2}m^{2}\gamma^{2}}{Dv^{2}}, \end{array}$$

which is the characteristic time required to damp momentum coherences over a distance *v*. The inverse $t_{d}^{-1}$ plays the role of a *decoherence* rate. Thus, the momentum space is the obvious choice for the preferred basis as already mentioned in [19].

### 3.2. Purity and Coherence Length in Momentum Space

As is known, a pure state can not be preserved along its open dynamics. This can be easily seen by evaluating the trace of the square of density matrix, ${\hat{\rho }}^{2}\left(t\right)$, or purity

(19) $$\begin{array}{c} \xi \left(t\right)=\int dp\int dp^{\prime }\left|\langle p\right|\hat{\rho }|p^{\prime }{\rangle |}^{2}=\int du\int dv{\left|\rho \left(u,v,t\right)\right|}^{2}. \end{array}$$

Writing Equations (11a) and (11b) in the form

**Figure 1:**
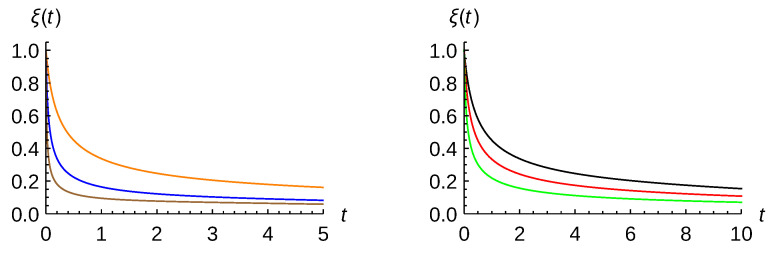
Purity ξ(t) given by Equation (21) for $\sigma_{0}=5$ and $k_{B}T=2$; and for minimum-uncertainty-Gaussian wavepacket with different values of the damping constant (**left** panel) and for γ=0.005 but with different values of the stretching parameter (**right** panel). Color curve codes are in the left panel: γ=0.01 (orange), γ=0.05 (blue), γ=0.2 (brown), whereas, in the right panel: η=0 (black), η=1 (red) and η=2 (green).

{(20a)d0(v,t)=−d02(t)v2−id01(t)v(20b)d1(v,t)=−d10(t)−id11(t)v

where the new coefficients are very easily identified, one obtains

(21) $$\begin{array}{c} \xi \left(t\right)=\frac{1}{2\sqrt{4\;d_{2}\left(t\right)\;d_{02}\left(t\right)-{\left(d_{11}\left(t\right)\right)}^{2}}} \end{array}$$

for the Gaussian solution given by Equation (8). Then, from Equations (11a)–(11c), the purity ξ(t) is an independent quantity on $p_{0}$, $x_{0}$ and the field strength *g*. As expected, it becomes unity for γ=0. Expanding ξ(t) in powers of *t* yields

(22) $$\begin{array}{c} \xi \left(t\right)=1+\left[2\gamma -\frac{4\sigma_{0}^{2}\left(1+\eta^{2}\right)}{\hbar^{2}}D\right]t+O\left(t^{2}\right) \end{array}$$

whereas, in the long time limit, γt≫1, purity becomes zero. We should stress that this linear behavior at short times is a typical feature of a Markovian regime [8,9]. In spite of this result, there are proofs in favor of the quadratic behavior [27,28]. Invariance of the trace under cyclic permutation has to be used, which is questionable when the state space is infinite-dimensional. Furthermore, the quadratic behavior is also found when one considers the time evolution of the Wigner function in phase space using a von Neumann-like equation but with the Poisson bracket of Hamiltonian and the Wigner function instead of their commutator [29]. As an illustration, in Figure 1, the evolution of purity with time is plotted for different damping constants (left panel) and stretching parameters (right panel) for $\sigma_{0}=5$ and $k_{B}T=2$. This quantity decays faster with γ than with η. The same behavior is expected when increasing the temperature although it is not shown in this figure.

The range of spatial coherence in momentum space can also be quantified by the off-diagonal direction $p=-p^{\prime }$ [10]. From Equations (8), (20a) and (20b), one observes that the width of the Gaussian in this direction is

(23) $$\begin{array}{c} \mu \left(t\right)=\sqrt{\frac{d_{2}\left(t\right)}{2[4d_{2}\left(t\right)\;d_{02}\left(t\right)-{\left(d_{11}\left(t\right)\right)}^{2}]}} \end{array}$$

which can be interpreted as the *coherence length in momentum space* [9]. Interestingly enough, the ratio of this coherence length and the distribution width provides again the purity

(24) $$\begin{array}{ccc} \frac{\mu (t)}{w_{t}} & = & \xi (t) \end{array}$$

where we have used Equation (21). This reveals that purity ξ(t) can also be interpreted as a dimensionless measure of decoherence [9]. From Equations (11a)–(10c), one sees that μ(t) decreases with the stretching parameter η. By expanding up to the second order in *t*, one obtains

(25) $$\begin{array}{c} \mu \left(t\right)≃\frac{\hbar }{2\sigma_{0}}\left\{1-4\eta^{2}\frac{\sigma_{0}^{2}D}{\hbar^{2}}t+D\left[-\frac{2}{m\hbar }\eta +\frac{8\sigma_{0}^{2}\gamma }{\hbar^{2}}\left(-\gamma +\frac{\sigma_{0}^{2}}{\hbar^{2}}\left(4+3\eta^{2}\right)D\right)\eta^{2}\right]t^{2}\right\}. \end{array}$$

Note that, for minimum-uncertainty-product wavepackets i.e., η=0, there are no linear and square terms in time. At long times, the coherence length vanishes according to

(26) $$\begin{array}{c} \mu \left(t\right)≃2m\gamma \hbar \sqrt{\frac{2}{D\;t}}. \end{array}$$

### 3.3. The Cat State

Let us consider now the initial state as a superposition of two well separated wave packets in momentum space,

(27) $$\begin{array}{c} \phi_{0}\left(p\right)\;=\;N\left(\phi_{0a}\left(p\right)+\phi_{0b}\left(p\right)\right) \end{array}$$

N being the normalization constant. From Equation (27), the initial density matrix has the form

(28) $$\begin{array}{c} \rho \left(p,p^{\prime },0\right)\;=\;N^{2}\left(\rho_{aa}\left(p,p^{\prime },0\right)+\rho_{ab}\left(p,p^{\prime },0\right)+\rho_{ba}\left(p,p^{\prime },0\right)+\rho_{bb}\left(p,p^{\prime },0\right)\right) \end{array}$$

where $\rho_{ij}\left(p,p^{\prime },0\right)=\phi_{0i}\left(p\right)\phi_{0j}^{*}\left(p^{\prime }\right)$; *i* and *j* being *a* or *b*. Due to the linearity of the master Equation (3), one obtains again the evolution of each term of Equation (28) separately by using the method outlined above i.e., by assuming a Gaussian ansatz. Afterwards, these solutions are superposed to have the time dependent PD according to [17],

(29) $$\begin{array}{c} P\left(p,t\right)\;=\;N^{2}\left(P_{aa}\left(p,t\right)+P_{ab}\left(p,t\right)+P_{ba}\left(p,t\right)+P_{bb}\left(p,t\right)\right). \end{array}$$

By using the fact that $P_{ba}\left(p,t\right)=P_{ab}^{*}\left(p,t\right)$, one can write

(30) $$\begin{array}{c} P\left(p,t\right)\;=\;N^{2}(P_{aa}\left(p,t\right)+P_{bb}\left(p,t\right)+2|P_{ab}\left(p,t\right)\left|\cos\Theta \left(p,t\right)\right) \end{array}$$

where $|P_{ab}\left(p,t\right)|$ is the modulus of $P_{ab}\left(p,t\right)$ and Θ(p,t) its phase. Rewriting Equation (30) as the typical interference pattern expression [8]

(31) $$\begin{array}{c} P\left(p,t\right)\;=\;N^{2}\left(P_{aa}\left(p,t\right)+P_{bb}\left(p,t\right)+2\sqrt{P_{aa}\left(p,t\right)P_{bb}\left(p,t\right)}\;e^{\Gamma (t)}\cos\Theta \left(p,t\right)\right), \end{array}$$

one has that

(32) $$\begin{array}{c} \Gamma \left(t\right)\;=\;\log\frac{|P_{ab}\left(p,t\right)|}{\sqrt{P_{aa}\left(p,t\right)P_{bb}\left(p,t\right)}}, \end{array}$$

Γ(t) being the so-called decoherence function which is negative. The corresponding exponential function

(33) $$\begin{array}{c} a\left(t\right)\;=\;e^{\Gamma (t)} \end{array}$$

is called the coherence attenuation coefficient which quantifies the reduction of the interference visibility [30].

Let us assume that the two wavepackets $\phi_{0a}\left(p\right)$ and $\phi_{0b}\left(p\right)$ are stretched Gaussian functions, Equation (10), co-centered in position space, $x_{0a}=x_{0b}=0$, having the same stretching parameter η, width $\sigma_{0}$, and different kick momenta, $p_{0a}$ and $p_{0b}$.

### 3.4. Free Evolution

Then, the evolution of the cross term $\rho_{ab}\left(p,p^{\prime },0\right)=\phi_{0a}\left(p\right)\phi_{0b}^{*}\left(p^{\prime }\right)$ is given by the Gaussian ansatz (8) with

$$\begin{array}{ccc} d_{0,ab}\left(v,t\right) & = & -\frac{{\left(p_{0a}-p_{0b}\right)}^{2}\sigma_{0}^{2}}{2\hbar^{2}}\left(1+\eta^{2}\right)+\left[\frac{\left(p_{0a}-p_{0b}\right)\sigma_{0}^{2}}{\hbar^{2}}\left(1+\eta^{2}\right)-i\frac{\tau (t)}{2\hbar m}\left[\left(p_{0a}+p_{0b}\right)+i\left(p_{0a}-p_{0b}\right)\eta \right]\right]v \end{array}$$

(34) $$\begin{array}{ccc} & & -\left[\frac{\sigma_{0}^{2}}{2\hbar^{2}}\left(1+\eta^{2}\right)-\frac{\tau {\left(t\right)}^{2}}{8m^{2}\sigma_{0}^{2}}+\frac{\tau (t)}{2\hbar m}\eta -D\frac{3+e^{-4\gamma t}-4e^{-2\gamma t}-4\gamma t}{16\hbar^{2}m^{2}\gamma^{3}}\right]v^{2} \end{array}$$

(35) $$\begin{array}{ccc} d_{1,ab}\left(v,t\right) & = & \frac{1}{2}e^{-2\gamma t}\left[\left(p_{0a}+p_{0b}\right)+i\left(p_{0a}-p_{0b}\right)\eta \right]-i\left[\frac{\tau (t)}{m}\left(\frac{\hbar }{4\sigma_{0}^{2}}e^{-2\gamma t}+\frac{D}{\hbar }\tau \left(t\right)\right)+\frac{1}{2}e^{-2\gamma t}\eta \right]v \end{array}$$

Note that the additional subscript ab refers to the cross term $\rho_{ab}\left(p,p^{\prime },0\right)$. For the evolution of the remaining terms of (28), one just uses the corresponding momenta in Equations (34) and (35); the function $d_{2}\left(t\right)$ remains the same as Equation (11c).

### 3.5. Linear Potential

In the presence of the external linear potential $\hat{V}=mg\hat{x}$, the evolution of the cross term $\rho_{ab}\left(p,p^{\prime },0\right)=\phi_{0a}\left(p\right)\phi_{0b}^{*}\left(p^{\prime }\right)$ is given by the same Gaussian ansatz (8) with

$$\begin{array}{ccc} d_{0,ab}\left(v,t\right) & = & -\frac{{\left(p_{0a}-p_{0b}\right)}^{2}\sigma_{0}^{2}}{2\hbar^{2}}\left(1+\eta^{2}\right)+\left[\frac{\left(p_{0a}-p_{0b}\right)\sigma_{0}^{2}}{\hbar^{2}}-i\frac{\tau (t)}{2\hbar m}\left[\left(p_{0a}+p_{0b}\right)+i\left(p_{0a}-p_{0b}\right)\eta \right]+i\frac{t-\tau (t)}{2h\gamma }g\right]v \end{array}$$

(36) $$\begin{array}{ccc} & & -\left[\frac{\sigma_{0}^{2}}{2\hbar^{2}}\left(1+\eta^{2}\right)-\frac{\tau {\left(t\right)}^{2}}{8m^{2}\sigma_{0}^{2}}+\frac{\tau (t)}{2\hbar m}\eta -D\frac{3+e^{-4\gamma t}-4e^{-2\gamma t}-4\gamma t}{16\hbar^{2}m^{2}\gamma^{3}}\right]v^{2} \end{array}$$

(37) $$\begin{array}{ccc} d_{1,ab}\left(v,t\right) & = & \frac{1}{2}e^{-2\gamma t}\left[\left(p_{0a}+p_{0b}\right)+i\left(p_{0a}-p_{0b}\right)\eta \right]-mg\tau \left(t\right)-i\left[\frac{\tau (t)}{m}\left(\frac{\hbar }{4\sigma_{0}^{2}}e^{-2\gamma t}+\frac{D}{\hbar }\tau \left(t\right)\right)+\frac{1}{2}e^{-2\gamma t}\eta \right]v. \end{array}$$

Analogously, for the evolution of the remaining terms of Equation (28), one just uses the corresponding momenta in Equations (36) and (37). Again, the function $d_{2}\left(t\right)$ is given by the same Equation (11c).

### 3.6. Decoherence

If the initial state is now a superposition of two stretched Gaussian wave packets with the same width and located symmetrically around the origin of momenta

(38) $$\begin{array}{c} \phi_{0}\left(p\right)\;=\;N{\left(\frac{2\sigma_{0}^{2}}{\pi \hbar^{2}}\right)}^{1/4}\left\{\exp\left[-\left(1+i\eta \right)\frac{{\left(p-p_{0}\right)}^{2}\sigma_{0}^{2}}{\hbar^{2}}\right]+\exp\left[-\left(1+i\eta \right)\frac{{\left(p+p_{0}\right)}^{2}\sigma_{0}^{2}}{\hbar^{2}}\right]\right\} \end{array}$$

where the normalization constant N is

(39) $$\begin{array}{c} N\;=\;{\left\{2+2\exp\left[-\frac{2p_{0}^{2}\left(1+\eta^{2}\right)\sigma_{0}^{2}}{\hbar^{2}}\right]\right\}}^{-1/2}, \end{array}$$

one readily obtains

{(40a)Γ(t)=−8p02σ04(1+η2)ℏ2sinh(2γt)ℏ2γe−2γt+4Dσ02sinh(2γt)D(40b)Θ(p,t)=4p0γησ02p+mgτ(t)ℏ2γe−2γt+4Dσ02sinh(2γt)

for the decoherence function and phase, respectively. Equation (40a) shows that Γ(t)=0 for D=0 implying that the last term in Equation (3) is responsible for decoherence [31]. The stretching parameter η speeds up the decoherence process. The external linear force does not affect the decoherence process; only the interference pattern is shifted. Furthermore, from Equation (40b), it is apparent that the phase function is zero for η=0 i.e., the interference pattern is not observed for minimum-uncertainty-product-Gaussian wave packets. This behavior is expected to also occur in isolated systems obeying the Schrödinger equation,

(41) $$\begin{array}{c} \phi \left(p,t\right)\;=\;\langle p|\phi \left(t\right)\rangle =\langle p|\hat{U}\left(t\right)|\phi \left(0\right)\rangle =e^{-ip^{2}t/\left(2m\hbar \right)}\phi \left(p,0\right) \end{array}$$

where for simplicity we have considered free propagation. There is only an overall phase factor. Thus, one has |ϕ(p,t)|=|ϕ(p,0)| and from which $\Theta \left(p,t\right)=\eta \frac{4p_{0}\sigma_{0}^{2}}{\hbar^{2}}p$; since the two wavepackets are well separated in the *p*-space with no overlapping, the interference term is practically zero, $P_{aa}P_{bb}≃0$.

In the long times limit, the decoherence function reaches the asymptotic value

(42) $$\begin{array}{c} \Gamma_{\infty }\;\approx \;-\frac{p_{0}^{2}}{2\sigma_{p}^{2}} \end{array}$$

where $\sigma_{p}=\hbar /2\sigma_{0}$. In the negligible dissipation limit where the third term is on the right-hand side of Equation (3) is neglected, one has that

{(43a)Γ(t)≈−16p02σ04(1+η2)Dℏ2(ℏ2+8Dσ02t)t(43b)Θ(p,t)≈η4p0σ02(p+mgτ(t))ℏ2+8Dσ02t.

In this limit and for times $t\ll \sigma_{p}^{2}/D$, one can introduce the decoherence time defined as

(44) $$\begin{array}{c} \tau_{D}=\frac{\sigma_{p}^{4}}{\left(1+\eta^{2}\right)p_{0}^{2}D};\;\Gamma \left(t\right)\approx -\frac{t}{\tau_{D}} \end{array}$$

Note that one can get the same result *directly* from Equation (40a) in this short time limit.

**Figure 2:**
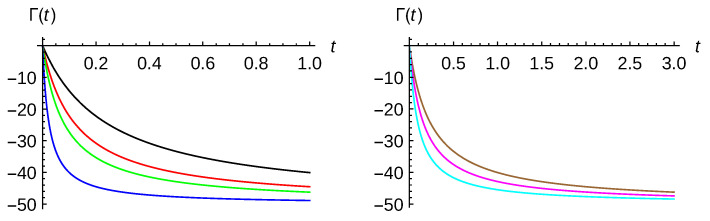
Decoherence function Γ(t) given by (40a) versus time for $k_{B}T=2$ (**left** panel) and for γ=0.005 (**right** panel). Color curve codes in the left panel are: γ=0.005 (black), γ=0.01 (red), γ=0.015 (green), γ=0.05 (blue); whereas in the right panel, $k_{B}T=2$ (brown), $k_{B}T=3$ (magenta) and $k_{B}T=5$ (cyan). Parameters for the two minimum-uncertainty Gaussian wave packets are $\sigma_{0}=5$ and $p_{0}=-1$.

As an illustration, in Figure 2, the decoherence function Γ(t), given by Equation (40a), is plotted versus time for $k_{B}T=2$ (left panel) and for γ=0.005 (right panel). In the left panel, the curves correspond to γ=0.005 (black), γ=0.01 (red), γ=0.015 (green), γ=0.05 (blue). In the right panel, the curves correspond to $k_{B}T=2$ (brown), $k_{B}T=3$ (magenta) and $k_{B}T=5$ (cyan). The initial parameters for the two minimum-uncertainty Gaussian (η=0) wavepackets are $\sigma_{0}=5$ and $p_{0}=-1$. In both cases, the asymptotic behavior is reached at relative small times. However, when varying the temperature, this behavior is reached around three times later. In other words, this function decreases faster with γ than with temperature $k_{B}T$.

## 4. Decoherence for Two-Identical-Particle Systems

Equation (1) is linear in $\hat{\rho }$. Writing it as $\dot{\hat{\rho }}=\hat{L}\hat{\rho }$, $\hat{L}$ being a linear operator and assuming ${\hat{\rho }}_{1}$ and ${\hat{\rho }}_{2}$ are two one-particle states describing two non-interacting particles 1 and 2, one can easily see that the time evolution for the product state ${\hat{\rho }}_{1}⊗{\hat{\rho }}_{2}$ is given by

(45) $$\begin{array}{c} \frac{\partial }{\partial t}\left({\hat{\rho }}_{1}⊗{\hat{\rho }}_{2}\right)\;=\;\left({\hat{L}}_{1}+{\hat{L}}_{2}\right)\left({\hat{\rho }}_{1}⊗{\hat{\rho }}_{2}\right). \end{array}$$

Let us consider now a system of two identical *spinless* particles. According to the spin-statistics theorem, the state of such a system must have a given symmetry under the exchange of particles; (anti-)symmetric for identical (fermions) bosons. By taking the initial momentum-space wavefunction as the pure state

(46) $$\begin{array}{c} \Phi_{\pm }\left(p_{1},p_{2},0\right)\;=\;N_{\pm }\left\{\phi \left(p_{1},0\right)\chi \left(p_{2},0\right)\pm \chi \left(p_{1},0\right)\phi \left(p_{2},0\right)\right\} \end{array}$$

ϕ and χ being one-particle wave functions and $N_{\pm }$ the normalization constant for bosons (+) and fermions (−), then the time evolution under the two-particle CL Equation (45) yields

(47) $$\begin{array}{ccc} \rho_{\pm }\left(p_{1},p_{2},p_{1}^{\prime },p_{2}^{\prime },t\right) & = & N_{\pm }^{2}\{\rho_{11}\left(p_{1},p_{1}^{\prime },t\right)\rho_{22}\left(p_{2},p_{2}^{\prime },t\right)+\rho_{22}\left(p_{1},p_{1}^{\prime },t\right)\rho_{11}\left(p_{2},p_{2}^{\prime },t\right)\\ & & \;\pm \rho_{12}\left(p_{1},p_{1}^{\prime },t\right)\rho_{21}\left(p_{2},p_{2}^{\prime },t\right)\pm \rho_{21}\left(p_{1},p_{1}^{\prime },t\right)\rho_{12}\left(p_{2},p_{2}^{\prime },t\right)\} \end{array}$$

where

{(48a)ρ11(p,p′,0)=ϕ0(p)ϕ0*(p′)(48b)ρ22(p,p′,0)=χ0(p)χ0*(p′)(48c)ρ12(p,p′,0)=ϕ0(p)χ0*(p′)(48d)ρ21(p,p′,0)=χ0(p)ϕ0*(p′).

Although $\rho_{11}\left(p,p^{\prime },t\right)$ and $\rho_{22}\left(p,p^{\prime },t\right)$ are one-particle densities, $\rho_{12}\left(p,p^{\prime },t\right)$ and $\rho_{21}\left(p,p^{\prime },t\right)$ are not. However, all of these functions are solutions of one-particle CL Equation (3) satisfying the continuity Equation (7). The joint detection probabilities are given by the diagonal elements of Equation (47);

(49) P±(p1,p2,t)=N±2[P11(p1,t)P22(p2,t)+P22(p1,t)P11(p2,t)±2Re{P12(p1,t)P21(p2,t)}]

where

(50) $$\begin{array}{c} P_{ij}\left(p,t\right)\;=\;\rho_{ij}\left(p,p,t\right) \end{array}$$

and the last term of Equation (49) is due to the symmetry of particles. In this context, and due to the environment, this term becomes zero over time, and we have decoherence in the sense of indistinguishability loss. Note that, for distinguishable particles obeying the Maxwell–Boltzmann (MB) statistics, the probability density is given by

(51) $$\begin{array}{c} P_{\mathrm{MB}}\left(p_{1},p_{2},t\right)\;=\;\frac{1}{2}\left[P_{11}\left(p_{1},t\right)P_{22}\left(p_{2},t\right)+P_{22}\left(p_{1},t\right)P_{11}\left(p_{2},t\right)\right]. \end{array}$$

For the single-particle density, $P_{\mathrm{sp},\pm }\left(p,t\right)=\int_{-\infty }^{\infty }dp_{2}\rho_{\pm }\left(p,p_{2};p,p_{2},t\right)$, one obtains

(52) Psp,±(p,t)=N±2[P11(p,t)+P22(p,t)±2Re{P12(p,t)s(t)}]

where the overlapping integral s(t) is

(53) $$\begin{array}{c} s\left(t\right)\;=\;\int_{-\infty }^{\infty }dp^{\prime }P_{21}\left(p^{\prime },t\right). \end{array}$$

Due to the continuity Equation (7), s(t) is a constant which does not depend on the environment parameters γ and *T* and time: $s\left(t\right)=\int dx^{\prime }P_{21}\left(p^{\prime },t\right)=\int dp^{\prime }P_{21}\left(p^{\prime },0\right)=\langle \chi \left(0\right)|\phi \left(0\right)\rangle$. On the other hand, if the system is isolated, states evolve under the Schrödinger equation and we have

(54) Psp,±(p,t)=N±2[|ϕ(p,t)|2+|χ(p,t)|2±2Re{〈χ(0)|ϕ(0)〉ϕ*(p,t)χ(p,t)}].

A comparison of Equations (52) and (54) reveals that, for open systems, the quantity $P_{12}\left(p,t\right)$ plays the role of $\phi^{*}\left(p,t\right)\chi \left(p,t\right)$. Thus, in analogy to Equation (31), we have again

(55) $$\begin{array}{c} |P_{12}\left(p,t\right)|\;=\;\sqrt{P_{11}\left(p,t\right)P_{22}\left(p,t\right)}e^{\Gamma_{12}\left(t\right)} \end{array}$$

leading to

(56) $$\begin{array}{c} \Gamma_{12}\left(t\right)\;=\;\log\frac{|P_{12}\left(p,t\right)|}{\sqrt{P_{11}\left(p,t\right)P_{22}\left(p,t\right)}}. \end{array}$$

By considering now one-particle states χ and ϕ as *minimum*-uncertainty-product Gaussian wave packets i.e., as in Equation (10) η=0, with parameters $y_{0}=0$, $q_{0}$, $\delta_{0}$ and $x_{0}=0$, $p_{0}$, $\sigma_{0}$, respectively, one obtains

(57) $$\begin{array}{ccc} P_{12}\left(p,t\right) & = & \sqrt{\frac{2\sigma_{0}\delta_{0}}{\sigma_{0}^{2}+\delta_{0}^{2}}}\frac{1}{\sqrt{4\pi b_{2}\left(t\right)}}\exp\left[b_{0}-\frac{{\left(p-b_{1}\left(t\right)\right)}^{2}}{4b_{2}\left(t\right)}\right] \end{array}$$

(58) $$\begin{array}{ccc} s(t) & = & e^{b_{0}}\;\sqrt{\frac{2\sigma_{0}\delta_{0}}{\sigma_{0}^{2}+\delta_{0}^{2}}} \end{array}$$

(59) $$\begin{array}{ccc} N_{\pm } & = & {\left\{2\left(1\pm \sqrt{\frac{2\sigma_{0}\delta_{0}}{\sigma_{0}^{2}+\delta_{0}^{2}}\exp\left[-\frac{{\left(p_{0}-q_{0}\right)}^{2}\sigma_{0}^{2}\delta_{0}^{2}}{\hbar^{2}\left(\sigma_{0}^{2}+\delta_{0}^{2}\right)}\right]}\right)\right\}}^{-1/2} \end{array}$$

where

(60) $$\begin{array}{ccc} b_{0} & = & -\frac{\sigma_{0}^{2}\delta_{0}^{2}}{\sigma_{0}^{2}+\delta_{0}^{2}}\frac{{\left(p_{0}-q_{0}\right)}^{2}}{\hbar^{2}} \end{array}$$

(61) $$\begin{array}{ccc} b_{1}\left(t\right) & = & e^{-2\gamma t}\frac{p_{0}\sigma_{0}^{2}+q_{0}\delta_{0}^{2}}{\sigma_{0}^{2}+\delta_{0}^{2}}-mg\tau \left(t\right) \end{array}$$

(62) $$\begin{array}{ccc} b_{2}\left(t\right) & = & e^{-4\gamma t}\frac{\hbar^{2}}{4(\sigma_{0}^{2}+\delta_{0}^{2})}+D\frac{1-e^{-4\gamma t}}{4\gamma }. \end{array}$$

Note that, for $\delta_{0}=\sigma_{0}$, one has $b_{2}\left(t\right)=w_{t}^{2}/2$. $P_{11}\left(p,t\right)$ and $P_{22}\left(p,t\right)$ are given by Equation (13) by using appropriate momenta. For $\delta_{0}=\sigma_{0}$ from Equation (56), one obtains

(63) $$\begin{array}{c} \Gamma_{12}\left(t\right)\;=\;-\frac{\sigma_{0}^{2}{\left(p_{0}-q_{0}\right)}^{2}}{2\hbar^{2}}\left\{1-{\left[1+D\frac{2\sigma_{0}^{2}}{\hbar^{2}\gamma }\left(e^{4\gamma t}-1\right)\right]}^{-1}\right\}. \end{array}$$

One sees that the decoherence function is negative and the same for both bosons and fermions. The decoherence process due to the last term of Equation (52) is interpreted here as loss of being indistinguishable as described in [17]. Notice that the case $p_{0}=q_{0}$ can take place only for bosons for which then the wave function (46) takes the product form just as classical states, revealing that quantum statistics is unimportant when the decoherence function $\Gamma_{12}\left(t\right)$ becomes zero. Another possibility for vanishing the last term of Equation (52) is when the overlapping integral is negligible. In such a case, the quantum statistics is unimportant too. However, this possibility can also happen in isolated systems, and it is not a result of interaction with the environment. Therefore, one should consider the effect of environment on $P_{12}\left(p,t\right)$ and $P_{21}\left(p,t\right)$ as an additional source of decoherence taking place for identical particle systems.

Decoherence can also be studied through what is called simultaneous detection probability i.e., measuring the joint detection probability for both particles in a given interval of the *p*-space. If we consider a detector, in momentum space, located at the origin with a width Δ, then the *ratio* of simultaneous detection probability of indistinguishable particles to the distinguishable ones is given by

(64) $$\begin{array}{c} p_{\pm }\left(t\right)\;=\;\frac{p_{\begin{array}{c} \mathrm{BE}\\ \mathrm{FD} \end{array}}\left(t\right)}{p_{\mathrm{MB}}\left(t\right)}=\frac{\int_{-\Delta /2}^{\Delta /2}dp_{1}\int_{-\Delta /2}^{\Delta /2}dp_{2}\;P_{\pm }\left(p_{1},p_{2},t\right)}{\int_{-\Delta /2}^{\Delta /2}dp_{1}\int_{-\Delta /2}^{\Delta /2}dp_{2}\;P_{\mathrm{MB}}\left(p_{1},p_{2},t\right)} \end{array}$$

where ± corresponds to bosons (Bose–Einstein statistics) and fermions (Fermi–Dirac statistics), respectively.

## 5. Results and Discussion

**Figure 3:**
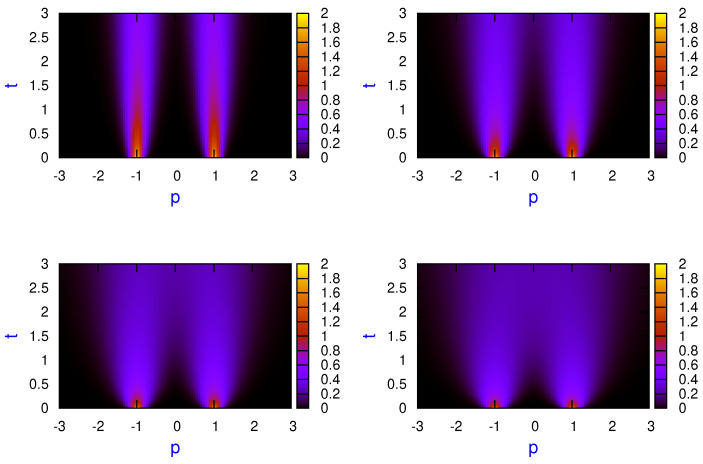
Probability density plots (31) for the superposition of two minimum-uncertainty-Gaussian wave packets, η=0, for γ=0.005 and different values of temperature: $k_{B}T=2$ (**left top** panel), $k_{B}T=5$ (**right top** panel), $k_{B}T=10$ (**left bottom** panel) and $k_{B}T=15$ (**right bottom** panel). The same parameters as in Figure 2.

Numerical calculations are carried out in a system of units where m=ℏ=1. In Figure 3, probability densities (31) together with Equations (40a) and (40b) are plotted for the cat state of two minimum-uncertainty-Gaussian wave packets, η=0, for γ=0.005 and different values of temperature: $k_{B}T=2$ (left top panel), $k_{B}T=5$ (right top panel), $k_{B}T=10$ (left bottom panel) and $k_{B}T=15$ (right bottom panel). The initial parameters used are the same as in Figure 2. As discussed previously, no interference pattern is observed in the momentum space at any temperature. Obviously, the width of the probability density also increases with time.

**Figure 4:**
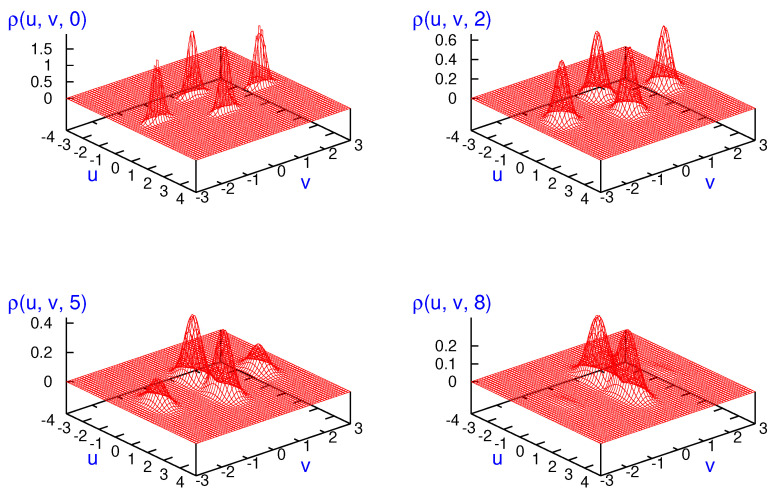
Density plots of modulus of density matrix elements given by time evolution of (28), |ρ(u,v,t)|, in uv-plane at different times t=0 (**left top** panel), t=2 (**right top** panel), t=5 (**left bottom** panel) and t=8 (**right bottom** panel) for η=0, g=0, γ=0.005 and $k_{B}T=2$, the same parameters as in Figure 2.

In order to gain some insight on this open dynamics, information about the reduced denrity matrix in the uv-plane is helpful. Thus, in Figure 4, density plots in the uv-plane at different times are shown for the cat state consisting of two minimum-uncertainty-product Gaussian wavepackets in the absence of external potential. The off-diagonal matrix elements |ρ(u,v,t)| are shown at t=0 (left top panel), t=2 (right top panel), t=5 (left bottom panel) and t=8 (right bottom panel) for η=0, g=0, γ=0.005 and $k_{B}T=2$. It is clearly seen how the coherences or off-diagonal matrix elements goes to zero at long times. The same behavior is observed when the stretching parameter is different from zero as well as the linear potential is present, this decoherence process being a little bit faster.

**Figure 5:**
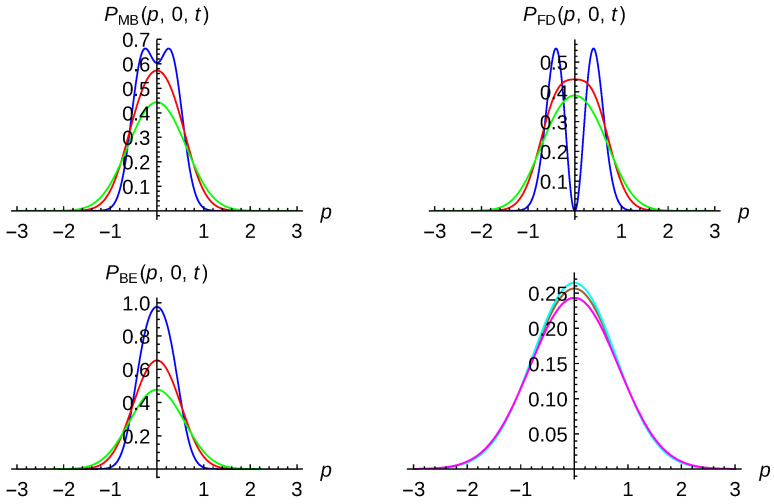
Two-particle probability density plots for finding a particle with zero momentum (51) for two distinguishable particles obeying MB statistics (**left top** panel) and (49) for two identical bosons (BE statistics, **left bottom** panel) and fermions (FD statistics, **right top** panel) at different times: t=0 (blue curves), t=1 (red curves) and t=2 (green curves). Right bottom panel depicts two-particle probability density for distinguishable particles (brown curve), identical bosons (cyan curve) and identical fermions (magenta curve) at t=5. One-particle states are taken as minimum-uncertainty-Gaussian wavepackets with the same width $\sigma_{0}=\delta_{0}=2$ and opposite kick momenta $p_{0}=-0.3$ and $q_{0}=0.3$. Environment parameters have been chosen to be γ=0.005 and $k_{B}T=5$.

Decoherence for identical particle systems discussed in previous section can be analyzed in several ways. First, in Figure 5, two-particle probability density plots for finding a particle with zero momentum and the second one at any value are shown. These results are issued from Equation (51) for two distinguishable particles obeying the MB statistics (left top panel) and Equation (49) for two identical bosons (left bottom panel) and fermions (right top panel) at different times: t=0 (blue curves), t=1 (red curves) and t=2 (green curves). The right bottom panel depicts the same two-particle probability density for distinguishable particles (brown curve), identical bosons (cyan curve) and identical fermions (magenta curve) at t=5. One-particle states are taken as minimum-uncertainty-Gaussian wave packets with the same width $\sigma_{0}=\delta_{0}=2$ and opposite kick momenta $p_{0}=-0.3$ and $q_{0}=0.3$. The parameters of the environment have been chosen to be γ=0.005 and $k_{B}T=5$. As can be seen, for distinguishable particles obeying the MB statistics, the two lobes at t=0 describes the two initial separated Gaussian wave packets. With time, the corresponding Gaussian wavepackets broaden and the two lobes disappear; the maximum being also around to zero momentum for the second particle. For bosons and fermions, the dynamics are quite different. The normalization factor also plays an important role since, from (59), one has that $N_{+}<N_{MB}<N_{-}$ where $N_{MB}=1/\sqrt{2}$. According to Equation (59), for our parameters, $N_{+}\approx 0.63$ and $N_{-}\approx 0.81$. The blue curves in each case display different behavior. At the initial time, bosons display a bunching-like behavior and fermions a clear anti-bunching like behavior, compared to distinguishable particles. For this open dynamics, the last term of Equation (49) together with the overlapping integral s(t) governs clearly the time evolution. The decoherence process takes place at t∼10 which is at least one order of magnitude less than the relaxation time $t_{r}=1/\gamma$. It should be emphasized that the decoherence time depends strongly on the choice of the one-particle states parameters. For instance, for motionless Gaussian wave packets with different widths $\sigma_{0}=3$ and $\delta_{0}=0.1$ where $N_{+}\approx 0.68$ and $N_{-}\approx 0.73$, the decoherence process takes place close to the relaxation time. With time, the initial bunching and anti-bunching character of the initial distributions are not preserved. Finally, in the right bottom panel, the two-particle probability density for the three kind of particles is plotted at t=5. As expected, the time behavior for the three types of particles is quite similar by starting from quite different initial conditions. Notice, however, that the fermionic distribution is a little bit broader than the distinguishable, and this is similar to the bosonic one. This can be seen as a reminder of the bunching and anti-bunching character of the initial distributions.

**Figure 6:**
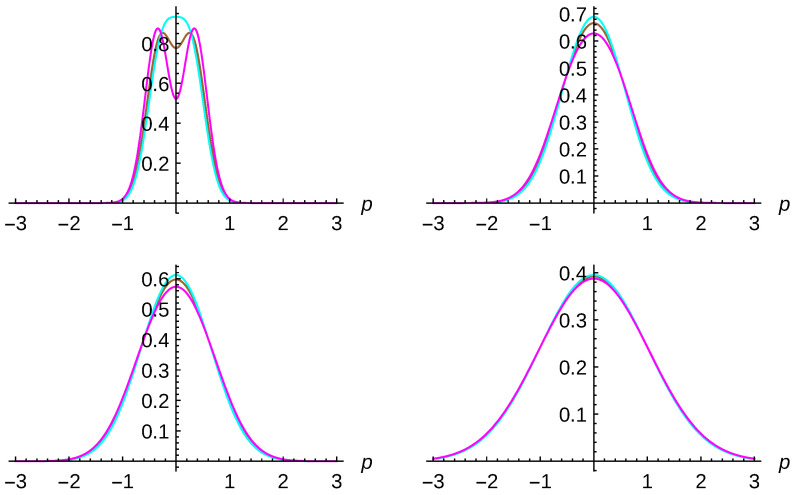
Single-particle probability density for distinguishable particles (brown curves); and for identical bosons (cyan curves) and fermions (magenta curves) at different times t=0 (**left top** panel), t=2 (**right top** panel), t=3 (**left bottom** panel) and t=10 (**right bottom** panel). One-particle states are taken as minimum-uncertainty-Gaussian wavepackets with the same widths $\sigma_{0}=\delta_{0}=2$ but opposite momenta $p_{0}=-0.3$ and $q_{0}=0.3$. Environment parameters have been chosen to be γ=0.005 and $k_{B}T=5$.

The second analysis one can carry out is on the single-particle probability. We can ask ourselves which is the corresponding probability density for finding a particle with momentum *p* independent on the momentum value of the second particle, see Equation (52). This is shown in Figure 6 for distinguishable particles (brown curves), identical bosons (cyan curves) and fermions (magenta curves) at different times t=0 (left top panel), t=2 (right top panel), t=3 (left bottom panel) and t=10 (right bottom panel). One-particle states are taken as minimum-uncertainty-Gaussian wavepackets with the same widths $\sigma_{0}=\delta_{0}=2$ but opposite momenta $p_{0}=-0.3$ and $q_{0}=0.3$. Environment parameters have been chosen to be γ=0.005 and $k_{B}T=5$. For these parameters, the decoherence time is one order of magnitude less than the relaxation time. The same behavior is observed with respect to the previous figure.

**Figure 7:**
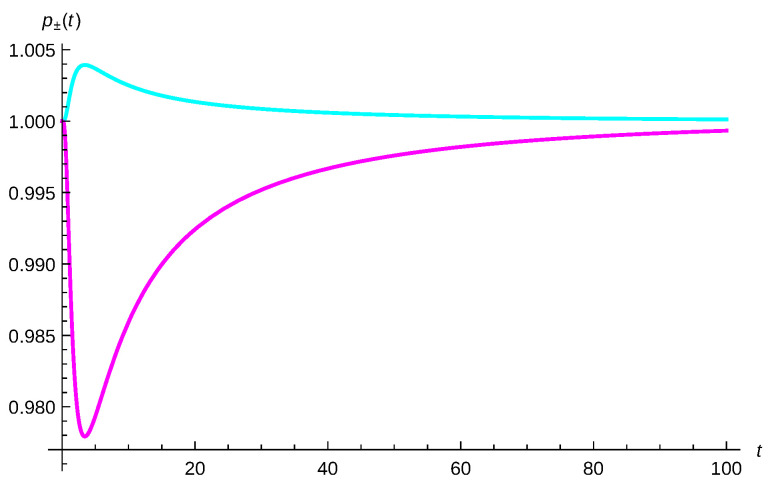
Relative simultaneous detection probability $p_{+}\left(t\right)=\frac{p_{BE}\left(t\right)}{p_{MB}\left(t\right)}$ (cyan) for two identical bosons and $p_{-}\left(t\right)=\frac{p_{FD}\left(t\right)}{p_{MB}\left(t\right)}$ (magenta) for two identical fermions, measured by a detector with a width Δ=2 located at the origin, see Equation (64). One-particle states are taken as minimum-uncertainty-Gaussian wavepackets with the same widths $\sigma_{0}=\delta_{0}=2$ but opposite momenta $p_{0}=-0.3$ and $q_{0}=0.3$ and the damping constant has been chosen to be γ=0.005.

The third type of analysis is by considering the simultaneous detection probability given by Equation (64). In Figure 7, the relative simultaneous detection probability $p_{+}\left(t\right)=\frac{p_{BE}\left(t\right)}{p_{MB}\left(t\right)}$ (cyan) for two identical bosons and $p_{-}\left(t\right)=\frac{p_{FD}\left(t\right)}{p_{MB}\left(t\right)}$ (magenta) for two identical fermions, measured by a detector with a width Δ=2 located at the origin are plotted. One-particle states are taken to be minimum-uncertainty-Gaussian wavepackets with the same widths $\sigma_{0}=\delta_{0}=2$ but opposite momenta, $p_{0}=-0.3$ and $q_{0}=0.3$, and the damping constant has been chosen to be γ=0.005. Using Equations (49) and (51) in (64) yields

(65) $$\begin{array}{c} p_{\pm }\left(t\right)\;=\;2N_{\pm }^{2}\left\{1\pm \frac{{\left|\int_{-\Delta /2}^{\Delta /2}dp\;P_{12}\left(p,t\right)\right|}^{2}}{\int_{-\Delta /2}^{\Delta /2}dp\;P_{11}\left(p,t\right)\int_{-\Delta /2}^{\Delta /2}dp\;P_{22}\left(p,t\right)}\right\}. \end{array}$$

In this figure, we can clearly see that, at short times, the symmetry of the corresponding wave functions is patent but at asymptotic times the simultaneous detection probability tends to one; that is, to the classical or MB statistics for both bosons and fermions.

Finally, in this work, we have put forward evidence of the quite different behavior of the decoherence process in the momentum space when considering cat states and identical spinless particles—whereas, with the first states, no diffraction pattern is observed in this space when compared with the configuration space for minimum uncertainty product Gaussian wave packets, a residual manifestation of the well-known bunching and anti-bunching properties of bosons and fermions is observed with time. This behavior is washed out more rapidly when increasing the damping constant and temperature.

## Data Availability

Not applicable.
